# Gene expression and protein secretion during human mesenchymal cell differentiation into adipogenic cells

**DOI:** 10.1186/s12860-014-0046-0

**Published:** 2014-12-20

**Authors:** Paola Romina Amable, Marcus Vinicius Telles Teixeira, Rosana Bizon Vieira Carias, José Mauro Granjeiro, Radovan Borojevic

**Affiliations:** Excellion Biomedical Services S.A., Rua Afrânio de Mello Franco 333, Quitandinha, Petrópolis, Rio de Janeiro Brazil; National Institute of Metrology, Quality and Technology (Inmetro), Xerém Rio de Janeiro, Brazil; Faculdade de Medicina de Petrópolis, Faculdades Arthur Sá Earp Neto, Petrópolis, Rio de Janeiro Brazil

**Keywords:** Human mesenchymal stromal cells, Adipogenesis, qPCR, Protein quantification

## Abstract

**Background:**

Mesenchymal stromal cells (MSC) can be obtained from potentially any tissue from the human body, but cells purified from different sources are undoubtedly different, and for each medical application, the MSC with the best regenerative potential should be chosen.

**Results:**

Bone marrow-derived mesenchymal stromal cells (BM-MSC), adipose tissue-derived mesenchymal stromal cells (AT-MSC) and Wharton’s Jelly-derived mesenchymal stromal cells (WJ-MSC) were isolated from human tissues and were cultured under differentiation media supplemented with fetal bovine serum. We quantified the expression of stem cell and adipocyte genetic markers using quantitative real time PCR, as well as the secretion of cytokines, extracellular matrix components and growth factors using Luminex and ELISA. All three MSC differentiated into adipogenic cells. AT-MSC showed the highest shift in *ADIPOQ*, *CEBPA* and *PPARG* mRNA expression. BM-MSC kept high expression levels of stem-cell markers *SOX2* and *POU5F1*. WJ-MSC showed the lowest increase in mRNA expression when cells were induced to differentiate into adipocytes. Regarding protein secretion, adipocyte-like cells generated from WJ-MSC secreted the highest chemokine levels. AT-MSC-derived adipocyte-like cells secreted the lowest cytokine amounts and the highest quantity of collagen types I and III. Adipocyte-like cells obtained from BM-MSC secreted high amounts of most angiogenic factors, growth factors TGF-β1 and TGF-β2, collagens type II and IV, heparan sulfate, laminin and aggrecan.

**Conclusion:**

Mesenchymal stromal cells purified from different tissues have a different behavior when induced to differentiate into adipocyte-like cells.

## Background

Adipose tissue has gained intense interest in the last decades. Early in the 19 s, adipose tissue was considered an agglomeration of fat-storing adipocytes surrounded by connective tissue. Only in 1948, Shapiro and Wertheimer suggested that adipose tissue was metabolically active [1]. Since then, several studies have improved our knowledge of adipose tissue metabolic, endocrine and immunologic functions. Fully differentiated adipocytes were first isolated using collagenase and flotation in 1964 by Rodbell [2]. Early in the 1990s, the first evidence of adipose tissue secretory function was reported [3]. Adipocytes secrete a wide variety of proteins, including adipokines, a family of proteins exclusively secreted by adipocytes [4]. The first adipokine described in 1994 was leptin, which confirmed that fat tissue has an important endocrine function [3].

In 2001, Zuk and colleagues described the procedure to process lipoaspirates [5]. The same group published subsequently biochemical and molecular analysis of the adipose tissue-derived stem cells, confirming their differentiation capacity into different mesodermal cell types and the expression of specific genes [6]. The possibility of stem cell isolation from the easily accessible and abundant adipose tissue was relevant in the stem cell and regenerative medicine field.

Before researchers completely understood adipose tissue biology, fat was already used in cosmetic, plastic, and reconstructive surgeries. In 1893, Neuber performed the first clinical fat transplantation, using small pieces of autologous material for filling depressed scars [7]. Problems related to necrosis, cyst formation and reabsorption were usual, due to low revascularization, especially when large fat pieces were transplanted.

Autologous fat transplantation has several advantages: it results in a satisfactory tissue repair, avoids large incision scars, causes no immune host responses, and avoids use of prosthesis. Its major disadvantage is the apparently unpredictable survival of the transplanted fat, since variable resorption rates have been reported (10% to 90%; [8]). In order to obtain the desired final volume of implant, multiple interventions may be required [9]. Mature adipocytes are fragile cells, and a large part may suffer extensive lesions with a loss of membrane integrity and release of fat. Sydney Coleman developed in 1986 an improved method for fat transplantation that minimizes the stress during tissue collection, by recommending transplantation of small fat tissue fragments in multiple steps, in order to facilitate revascularization and, therefore, tissue survival. This tissue processing, called Coleman’s method, resulted in reduced graft resorption [10].

The discovery of stem cells in adipose tissue by Zuk and colleagues in 2001 complemented the understanding of the durability of fat transfer [5]. Matsumoto and coworkers developed in the year 2006 a new technique to reduce tissue resorption, using the “cell-assisted lipotransfer” [11]. The technique consisted in separating a part of the aspirated fat in a stromal vascular fraction enriched in MSC. The lipotransfer containing these cells allowed a higher survival and vascularization of the graft in pre-clinical and clinical trials [12]. Further studies have proposed alternative procedures to manipulate lipoaspirates, and overall recommendations for handling of samples and for characterization of transferred cells [12,13].

The MSC content in primary cell suspensions collected from lipoaspirates is however low. Immunophenotyping of cells in the stromal fraction indicates presence of blood-vessel components and associated elements, as well as pre-adipocytes and MSC. The latter two are characterized as CD45^neg^, CD34^pos^ and/or CD105^pos^, and their content in the stromal fraction was reported to be below 5% [14]. Rigotti and coworkers reported in the year 2007 that the content of MSC in the adipose tissue stromal fraction was in order of 1%, but importantly, the level of clonogenic colony-forming units that possibly better reflects the quantity of cells able to participate in long-term tissue regeneration was tenfold lower [15]. In contrast, when selected by plastic adhesion (after *in vitro* tissue digestion) and expanded *in vitro*, MSC are selected and can represent 70-90% of the harvested cells. The use of these cells offers new cues in regenerative procedures using much higher levels of MSC in the “cell-assisted lipotransfers”.

The MSC used in this procedure can be autologous, requiring a first harvest of fat tissue to establish cultures, and a second one to provide the tissue for the graft. When the donor adipose tissue is limited, alternative sources of MSC may be used, associated with autologous fat graft, in order to grant a long-term restoration of fat tissue under controlled conditions, maintaining the volume and the implant’s form. Among others, this may be the case of facial fat restoration in lipodystrophies or in chronic HIV infection [16-18]. We hypothesized that MSC from different origins other than adipose tissue can be used in assisting fat transplantation, especially if they show a high adipogenic differentiation potential. MSC obtained from the Wharton’s Jelly of the umbilical cord, a convenient source of cells with high proliferation capacity, may be proposed for allogeneic transplantation. Therefore, we characterized MSC obtained from different sources (adipose tissue, Wharton’s Jelly and bone marrow) regarding their ability to differentiate into adipocytes.

Characterization of MSC and their ability to differentiate into adipocytes may be relevant in many other fields of regenerative medicine. Novel proposals for tendon and muscle regeneration deal with the use of MSC for repair of such lesions of shoulder, which are particularly sensitive to the fatty degeneration of muscles [19]. This is generally an irreversible process, in which adipocytes invade and replace the muscle tissue. The origin of these adipocytes is a differentiation from resident MSC, which in the absence of mechanical stimulation by muscle contraction suffer a decrease of Wnt signaling, leading to transcription of adipogenic factors such as PPARγ, C/EBPα and leptin [20]. Knowledge of which MSC already express, or easily activate expression of adipogenic factors, and readily differentiate into adipocytes, may be relevant for the choice of MSC to be used in such a therapy.

In the present study, we compared adipogenic differentiation in vitro of MSC derived from bone marrow, adipose tissue and Wharton’s Jelly. Our main goal is to determine which source of MSC is more appropriate for allogeneic transplantation, so they can be use together with adipose tissue for long-lasting effects after fat transplantation. We quantified the gene expression of key pluripotent and differentiation markers, and the secretion of different proteins, and we compared these results with those recently obtained by our group regarding protein secretion of undifferentiated BM-MSC, AT-MSC and WJ-MSC.

## Results

MSC obtained from human bone marrow, adipose tissue and Wharton’s Jelly identity was confirmed: they adhered to the plastic surfaces of cell culture flasks with a fibroblast–like morphology, expressed the cell surface markers CD73, CD90 and CD105 and negative for hematopoietic markers (HLA-DR, CD45, CD14 and CD34) [15]. MSC also differentiated into all three mesodermal lineages: adipocytes, osteoblasts and chondroblasts (results shown in 24).

All MSC cultured in DMEM supplemented with FBS and with the compounds recommended for adipocyte induction differentiated into adipocyte-like cells. Differentiation controls maintained in the standard medium were run in parallel for all conditions, and they were all negative, not presenting any visible lipid accumulation (data not shown).

### Gene expression during differentiation into adipocyte-like cells

Gene expression of pluripotent (*SOX2*, *POU5F1* and *TERT*) and adipogenic (*PPARG*, *ADIPOQ* and *CEPBA*) markers were studied, evaluating cell differentiation effects on gene expression. Appropriate reference genes were *RPL13A* for WJ-MSC and AT-MSC, and *HPRT1* for BM-MSC [21].

*TERT* expression was not detected in any sample. Published data are contradictory regarding *TERT* expression: some authors described *TERT* mRNA as a pluripotent marker in BM-MSC [25] but many others detected neither *TERT* mRNA [26] nor TERT activity [27,28] in MSC primary cultures.

Data on mRNA expression during adipogenic differentiation are shown in Table 1. A statistical analysis was performed comparing the expression of one gene for all cells and time points evaluated. For all the genes, AT-MSC at day 21 expressed statistically significant higher amounts of the corresponding mRNA. Regarding pluripotent markers, only *POU5F1* was downregulated in BM-MSC cultures.

**Table 1:** **Relative gene expression (expressed in mean ± standard deviation) for human adipose tissue- (AT-), bone marrow- (BM-) and Wharton’s Jelly-mesenchymal stromal cells (WJ-MSC) when grown in low glucose media supplemented with 10% fetal bovine serum**

	**BM-MSC**		**AT-MSC**		**WJ-MSC**	
	**d10**	**d21**	**d10**	**d21**	**d10**	**d21**
*PPARG*	14.9 ± 2.5 ^a^	32.3 ± 3.0 ^a^	21.0 ± 6.4 ^a^	58.3 ± 13.6 ^b^	15.2 ± 8.9 ^a^	29.1 ± 3.2 ^a^
*ADIPOQ*	4,126.8 ± 3,370.2 ^a^	3,994.2 ± 1,292.7 ^a^	3,378.4 ± 2,243.0 ^a^	423,626.8 ± 147,352.8 ^b^	*	*
*CEBPA*	102.6 ± 14.6 ^a^	89.7 ± 33.1 ^a^	206.6 ± 53.9 ^a^	1,271.3 ± 245.3 ^b^	12.1 ± 6.6 ^a^	17.3 ± 4.3 ^a^
*SOX2*	**	2.5 ± 1.4 ^a^	7.1 ± 0.8 ^a^	4,800.8 ± 387.7 ^b^	5.8 ± 3.0 ^a^	3.0 ± 1.1 ^a^
*POU5F1*	0.7 ± 0.3 ^a^	0.6 ± 0.1 ^a^	4.0 ± 1.0 ^a^	142.5 ± 0.2 ^b^	3.9 ± 2.2 ^a^	0.3 ± 0.1 ^a^

*ADIPOQ*: adiponectin; AT-MSC: adipose tissue-derived MSC; BM-MSC: bone marrow-derived MSC; *CEPBA*: CCAAT/enhancer-binding protein alpha; d10: day 10; d21: day 21; MSC: mesenchymal stromal cells; *POU5F1*: POU domain, class 5, transcription factor 1; *PPARG*: peroxisome proliferator-activated receptor gamma; *SOX2*: SRY (Sex Determining Region Y)-Box 2; WJ-MSC: Wharton’s Jelly-derived MSC; *not detected in initial sample (d0); **not detected in that particular sample (d10 or d21).

Negative values mean downregulation on mRNA expression when compared to non-induced cultured cells, and positive values, upregulation. Statistical significance was assessed by one-way non-parametric ANOVA followed by Bonferroni post-hoc test to compare all pairs of data for a determined gene (α = 0.05, n = 3). In a same row, a same letter means that both conditions are not significantly different; different letters (superscript letters a and b) indicate statistically different values.

Regarding adipogenic markers, *ADIPOQ* was detected only at day 0 in WJ-MSC, but it showed the highest increase in AT-(day 21) and BM-MSC (day 10). Highest upregulation of adipogenic markers in BM-MSC and AT-MSC correlated with highest lipid accumulation in differentiation assays and with a lower expression levels of WJ-MSC.

### Protein secretion by adipocyte-like cells

Results from cell culture supernatant quantification of secreted chemokines, pro- and anti-inflammatory cytokines, angiogenic and growth factors, extracellular matrix components and matrix metalloproteinases are shown in Table 2. In order to make results comparable, supernatant concentration was expressed in pg secreted per million cells and per day, since MSC from different sources proliferated in significantly different rates when differentiated into the adipogenic phenotype. Final cell concentration was 26,000.90 cells/mL for BM-MSC, 123,333.4 cells/mL for AT-MSC and 296,875.0 cells/mL for WJ-MSC.

**Table 2:** **Protein secretion (expressed as mean ± standard deviation) of adipose tissue- (AT-), bone marrow- (BM-) and Wharton’s Jelly-mesenchymal stromal cells (WJ-MSC) when grown in low glucose media supplemented with 10% fetal bovine serum**

	**BM-MSC**	**AT-MSC**	**WJ-MSC**
**Chemokines (pg/10** ^**6**^ **cells/day)**			
**IP-10**	0.8 ± 0.3 ^a^	nd	3.5 ± 0.4 ^a^
**MCP-1**	727.5 ± 86.5 ^a^	336.2 ± 42.2 ^b^	551.5 ± 51.6 ^a,b^
**Pro-inflammatory cytokines (pg/10** ^**6**^ **cells/day)**			
**IL-6**	46.6 ± 2.8 ^a^	31.2 ± 4.3 ^a^	146.0 ± 31.6 ^b^
**IL-7**	102.4 ± 57.8 ^a^	0.13 ± 0.19 ^a^	0.07 ± 0.10 ^a^
**IL-8**	628.3 ± 8.0 ^a^	63.7 ± 2.7 ^a^	4,466.3 ± 634.6 ^b^
**IL-12**	41.5 ± 7.6 ^a^	1.6 ± 2.3 ^a^	nd
**Anti-inflammatory cytokines (pg/10** ^**6**^ **cells/day)**			
**IL-1ra**	30.00 ± 0.01 ^a^	nd	205.3 ± 28.7 ^a^
**Angiogenic factors (pg/10** ^**6**^ **cells/day)**			
**Angiogenin**	3,054.7 ± 74.2 ^a^	995.3 ± 34.9 ^b^	517.9 ± 5.3 ^c^
**Thrombospondin-2**	79,967.7 ± 149.8 ^a^	35,564.0 ± 1,644.6 ^b^	155,582.9 ± 6,467.8 ^c^
**PLGF**	102.4 ± 2.1 ^a^	12.5 ± 1.0 ^a^	nd
**aFGF**	65.7 ± 12.4 ^a^	6.9 ± 6.5 ^b^	14.0 ± 2.6 ^b^
**VEGF-D**	24.80 ± 0.01	nd	nd
**Endostatin**	1,322.3 ± 24.7 ^a^	1,270.4 ± 21.6 ^a^	2,343.3 ± 68.7 ^b^
**Angiopoietin-1**	129,196.1 ± 8,604.8 ^b^	12,502.3 ± 405.8 ^a^	17,114.5 ± 51.9 ^a^
**VEGF**	3,431.8 ± 180.9 ^b^	392.4 ± 24.4 ^a^	3.4 ± 0.8 ^a^
**Growth factors (pg/10** ^**6**^ **cells/day)**			
**TGF-β1**	4,051.7 ± 0.0 ^a^	1,844.7 ± 205.9 ^b^	1,622.6 ± 51.1 ^b^
**TGF-β2**	178.1 ± 1.1 ^a^	17.7 ± 3.3 ^b^	13.7 ± 3.7 ^b^
**HGF**	268.0 ± 8.4 ^a,b^	44.8 ± 8.6 ^a^	314.0 ± 86.6 ^b^
**G-CSF**	nd	nd	52.6 ± 5.9
**PDGF-AA**	7.9 ± 1.5 ^a^	2.5 ± 4.7 ^a^	6.9 ± 1.7 ^a^
**Extracellular matrix proteins (pg/10** ^**6**^ **cells/day)**			
**Collagen I**	76.6 ± 6.0 ^a^	313.2 ± 53.0 ^b^	15.6 ± 0.8 ^a^
**Collagen II**	2,210.4 ± 911.3	nd	nd
**Collagen III**	12,332.2 ± 0.0 ^a^	17,164.5 ± 689.8 ^a^	38,914.8 ± 477.9 ^b^
**Collagen IV**	116.5 ± 2.0 ^a^	51.9 ± 4.2 ^b^	nd
**Elastin**	959.3 ± 236.4 ^a^	300.1 ± 6.5 ^b^	nd
**Fibronectin**	nd	1.4 ± 1.2	nd
**Heparan sulfate**	235.6 ± 111.0 ^a^	27.5 ± 233.0 ^a^	nd
**Laminin**	95.5 ± 14.0 ^a^	42.2 ± 15.2 ^a^	nd
**Aggrecan**	2.7 ± 0.1 ^a^	0.2 ± 0.4 ^a^	nd
**Matrix metalloproteinases (pg/10** ^**6**^ **cells/day)**			
**MMP1**	149.6 ± 0.0 ^a,b^	284.2 ± 11.0 ^a^	364.0 ± 36.9 ^b^
**MMP3**	1,235.1 ± 0.0 ^a^	17,387.1 ± 3,213.2 ^b^	684.2 ± 49.9 ^a^
**MMP7**	675.6 ± 0.0 ^a,b^	102.9 ± 26.7 ^a^	35.5 ± 15.7 ^b^
**MMP8**	527.0 ± 152.2 ^a^	nd	10.6 ± 157.3 ^b^
**MMP13**	24,201.6 ± 124.7 ^a^	22.5 ± 0.0 ^b^	nd

aFGF: acidic fibroblast growth factor; AT-MSC: adipose tissue-derived MSC; BM-MSC: bone marrow-derived MSC; GCS-F: granulocyte-colony stimulating factor; HGF: hepatocyte growth facctor; IL: interleukin; IP-10: interferon-gamma induced protein 10; MCP-1: monocyte chemotactic protein 1; MMP: metalloproteinase; nd: not determined; PDGF-AA: platelet derived growth factor AA; pg: picogram; PLGF: placental growth factor; TGF: transforming growth factor; VEGF: vascular endothelial growth factor; WJ-MSC: Wharton’s Jelly-derived MSC.

Protein concentration is expressed in pg secreted per million cells and per day. ANOVA and Bonferroni tests (α = 0.5, n = 3) were applied for each quantified factor in order to determine significant differences between all three MSC. For a specific protein; different letters (superscript letters a, b and c) mean statistically different expression levels.

Only IP-10 and MCP-1 were detected in supernatants of cells induced to differentiate into adipocytes. IP-10 secretion was higher for WJ-MSC, and MCP-1 was higher for BM-MSC. AT-MSC secreted the lowest concentration of both chemokines.

Pro-inflammatory cytokines GM-CSF, IL-1β, TNF-α, IFN-γ, IL-2, IL-2R, IL-15 and IL-17 were not detected in any of the supernatants of differentiated cells. Only IL-6, IL-7, IL-8 and IL-12 were detected in all supernatants. Again, AT-MSC secreted the lowest concentration of all 4 detected cytokines. BM-MSC secreted higher amounts of IL-7 and IL-12, with concentration rates of 102.4 ± 57.8 and 41.5 ± 7.6 pg/10^6^ cells/day, respectively. On the other side, WJ-MSC secreted 146.0 ± 31.6 pg IL-6/10^6^ cells/day and 4,466.3 ± 634.6 pg IL-8/10^6^ cells/day, higher secretion rates when compared to BM- and AT-MSC.

In the group of anti-inflammatory cytokines, only IL-1RA was quantified in cell culture supernatants: the highest secretion rate was 205.3 ± 28.7 pg/10^6^ cells/day in WJ-MSC, but IL-1RA was undetectable in AT-MSC supernatants. IL-4, IL-5, IL-10, IL-13 and IFN-α were not detected in any supernatant.

All the studied angiogenic factors were found in culture supernatants. BM-MSC secreted the highest concentrations of angiogenin, PLGF, aFGF, VEGF-D, angiopoietin and VEGF. WJ-MSC showed the highest secretion rates for thrombospondin-2 and endostatin. PLGF was not detected in WJ-MSC supernatants and VEGF-D was only detected in BM-MSC cell cultures.

EGF, bFGF, IGF-1, TGF-β3, PDGF-AB and PDGF-BB were the only growth factors that were not detected in adipocyte-like cell supernatants. G-CSF was only detected in WJ-MSC, which also secreted the highest amounts of HGF (314.0 ± 86.6 pg/10^6^ cells/day), as compared to secretion rates of AT-MSC and BM-MSC. BM-MSC showed the highest secretion rates of PDGF-AA (7.9 ± 1.5 pg/10^6^ cells/day) and both detected TGF forms, β1 (4,051.7 ± 0.0 pg/10^6^ cells/day) and β2 (178.1 ± 1.1 pg/10^6^ cells/day).

Decorin and perlecan were not detected in any cell culture supernatant. Only BM-MSC secreted collagen type II and only AT-MSC secreted fibronectin in detectable concentrations. WJ-MSC showed a poor extracellular matrix production, since several proteins were not detected in their supernatants (fibronectin, collagen type II and type IV, heparin sulfate, laminin and aggrecan). WJ-SC secreted the highest amounts of collagen type III, AT-MSC secreted the highest amounts of collagen type I, and BM-MSC collagen type IV. BM-MSC secreted highest heparan sulfate, laminin, aggrecan and elastin.

All the matrix metalloproteinases were detected in adipocyte-like cell supernatants, except for MMP13 in WJ-MSC and MMP-8 in AT-MSC. MMP-1 was secreted in higher concentrations by WJ-MSC, which did not secrete MMP-13. We could not detect MMP-8 in AT-MSC supernatants, but these cells where the best secreting-cells of MMP-3. BM-MSC were the best secreting cells regarding MMP-7, MMP-8 and MMP-13.

## Discussion

The present study was performed in order to compare the MSC derived from bone marrow, adipose tissue and Wharton’s Jelly along the *in vitro* differentiation into the adipocyte-like phenotype, in terms of gene expression and protein secretion into the culture supernatant. The major objective was improving the potential protocols for the “cell-assisted lipotransfer”, obtaining a more efficient and durable gain in volume of the transplanted tissue. In general terms, we wanted also to check differences among MSC from three standard sources, often used in regenerative therapies, when submitted to the same biological induction of differentiation. Within these tissues, the MSC populations have different compositions and specific functions, which may be partially maintained during in vitro expansion.

Bone marrow is a traditional source of MSC, in which the first mesenchymal stem cells giving rise to fibroblastoid colonies were described [29]. BM-MSC have multiple functions, notably the maintenance of the osteogenic cell pool [30,31] and the provision of the stroma that sustains hematopoiesis [31]. In the latter group (BM-MSC differentiating into stromal cells that gives support to hematopoiesis) are found bone marrow fat-storing cells, which accumulate lipids in multilocular droplets, but have several physiological differences as compared to the typical adipose tissue adipocytes. Bone marrow also harbors multipotent mesenchymal stem cells that can be mobilized to the peripheral circulation by chemokines [32], and participate in repair and regeneration in distant organs [33].

Adipose tissue MSC are associated with the vascular stroma, where they participate in the maintenance of blood and lymphatic vessels [34]. In the same location, they can give rise to preadipocytes, already engaged in adipocyte differentiation and expressing the major master genes of this pathway, but without accumulation of fat [35].

Wharton’s Jelly contains MSC that are not necessarily associated with vessels and have essentially a mechanical function [36]. Due to the easy access to this normally discarded tissue, the broad capacity of proliferation and the capacity of WJ-MSC to respond to differentiation induction [37,38], these cells are considered to be a rich source of cells for allogeneic transplantation protocols.

As expected, the present study has shown broad differences among the MSC behavior during the induction of the adipocyte-like differentiation. Regarding gene expression, AT-MSC showed the higher shift in the expression of adipogenic-related genes (*ADIPOQ*, *PPARG* and *CEBPA*), when comparing expression after culturing cells in adipogenic media against baseline expression (mRNA detected in cells cultured in parallel in control media). The lipid accumulation was more intense and rapid in AT-MSC as compared to other MSC. BM-MSC were able to deposit lipids in droplets (stained with Oil Red) of similar size as those found in AT-MSC, but the amount of lipid deposits was lower (as judged per microscopic visualization; data not shown). In contrast, WJ-MSC showed a more immature differentiation state at the end of the induction period (21 days), with few lipid vacuoles and little lipid accumulation. Similar results regarding lower adipogenic potential of WJ-MSC were reported previously by other authors [38,39].

Secretion profile of the adipocyte-like cells isolated from the three tissues would be expected to be similar, considering that they were differentiated into the same cell type, but behaviors were very different among all cells. Chemokines IP-10 and MCP-1 were found in adipocyte-like supernatants, and secretion rates were different among the three MSC sources. Gerhardt and colleagues described in the year 2001 MCP-1 secretion by pre-adipocytes and correlated it with macrophage recruitment [40]; they demonstrated that MCP-1 secretion is up-regulated under inflammatory stimulation (TNF-α) and that secretion is reduced when pre-adypocytes are fully differentiated into adipocytes. Therefore, lower MCP-1 secretion by adipocyte-like cells derived from AT-MSC would indicate a more advanced differentiation stage. Xu and colleagues performed *in vivo* studies in obese mice and hypothesized that after a certain threshold of obesity, macrophage recruitment (through MCP-1 secretion) is improved, resulting in a feedback loop where insulin signaling is impaired, leading to adipocyte lipolysis and necrosis [41]. Human adipocyte secretion of IP-10 was also confirmed and was positively correlated with body mass index [42].

IL-6 and IL-7 secretion by adipocytes was confirmed in 1997 and 2007, respectively, and plasmatic concentrations of both cytokines were positively correlated with obesity [43,44] Regarding IL-8, results are not conclusive: Okada and coworkers reported that IL-8 secretion was augmented after BM-MSC adipogenesis [45], similar to what we obtained, if we compare secretion of adipocyte-like cells derived from BM-MSC and basal secretion (47.8 ± 11.7 versus 628.3 ± 8.0 pg/10^6^ cells/day; 24). On the other side, Fain reported in 2006 that non-fat cells of adipose tissue secrete higher concentrations of IL-8, when compared with adipocytes [46]. These results are in agreement with what we are reporting here: undifferentiated AT-MSC showed a higher IL-8 secretion rate than adipocyte-like cells derived from these cells [24]. When looking at IL-12, Blaber and coworkers found that IL-12 secretion was higher in AT-MSC than in adipocytes, results that agree with what we found for AT- and WJ-MSC, but not for BM-MSC [46]. As we reported here, IL-5 was not detected in supernatants of human adipocytes [47].

While we did not detected IFN-γ, IL1β, IL17, TNFα, IL4, IL13, IL10, MIP1α, bFGF, GM-CSF and IL2 in adipocyte-like cell supernatant, Blaber and colleagues also reported low concentrations of these cytokines in adipocyte supernatants, suggesting a difference in secretome composition when comparing mature adipocytes with adipocyte-like cells obtained by differentiation induction of MSC [47].

All angiogenic factors were expressed by adipocyte-like cells and by the MSC as well [24]. Already during embyogenesis, angiogenesis and adipogenesis are strongly related and it was previously demonstrated that AT-MSC, pre-adipocytes and adipocytes secrete a variety of angiongenic factors (reviewed in [48]). Pro-inflammatory cytokines are also involved in angiogenesis, since they attract cells that also are able to secrete pro-angiogenic factors. Angiogenesis is highly desirable during fat transplantation, since new vasculature would allow tissue survival and less reabsortion. Therefore, adipocyte-like cells derived from BM-MSC would be a good supplement for adipose tissue during transplantation.

Metalloproteinases (MMPs) are essential during angiogenesis and consequently during adipose tissue development. Adipose tissue secretes several MMPs, like MMP-3 and −13, and downregulation was observed for other MMPs, like MMP-7 (reviewed in [48]). Neither WJ-MSC nor adipocyte-like cells from WJ-MSC secreted MMp-13 in detectable concentrations; the same for MMP-8 in AT-MSC and adipocytes differentiated from them [24]. BM-MSC augmented MMP-3 and −13 secretion after differentiation, again indicating suitability for fat transplantation supplementation. MMP-3 degrades several types of extracellular matrix proteins, like collagen types II, III, IV, IX, and X, proteoglycans, fibronectin, laminin, and elastin and can also activate other MMPs, such as MMP-1, MMP-7, and MMP-9.

Growth factors found in adipocyte-like cells were G-CSF, HGF, PDGF-AA and TGF-β1 and β2. Rahimi and colleagues reported in 1998 that pre-adipocytes and adipocytes secreted both TGF-β1 and β2 and that these growth factors negatively affected adipocyte differentiation [49]. Our results showed an increased TGF secretion rate for all MSC differentiating into adipocyte-like cells, except for TGF-β2 in adipocyte-like cells derived from WJ-MSC. No information regarding PDGF-AA secretion by adipocytes was found, but since this molecule is a potent mitogen for MSC, its secretion is considered positive for fat transplantation. Results reported before by our group showed that non-induced BM-, AT- and WJ-MSC secreted PDGF-BB. In the present study, after adipogenic differentiation PDGF-BB was not detected in any supernatant, indicating a downregulation when cells enter the adipogenic differentiation pathway [24].

Collagen type IV is the most abundant collagen type in adipose tissue and its absence had been linked with obesity [50]. Considering our results, WJ-MSC would not be appropriate cells for fat transplantation, since neither the undifferentiated cells nor the adipocyte-like cells derived from them secreted detectable concentrations of collagen type IV. On the other side, BM-MSC did not secreted detectable concentrations of collagen type IV while undifferentiated but adipogenic media stimulated its secretion at a rate of 116.5 ± 2.0 pg/10^6^ cells/day. Nakajima and coworkers reported that laminin and fibronectin are also important components of adipose tissue extracellular matrix, while collagen type II was found in an extremely low amount [51]. Fibronectin was not detected in any MSC supernatant [24] and after adipogenic differentiation, only adipocyte-like cells derived from AT-MSC secreted it in detectable concentrations. Laminin was secreted by BM- and AT-MSC in both, differentiated and undifferentiated states, but no laminin was detected in WJ-MSC supernatants. Collagen type II was only quantified in adipocytes derived from BM-MSC. Another important result we obtained here is that adipocyte-like cells derived from WJ-MSC were poor producers of extracellular matrix components and this correlated with results reported before by our group where undifferentiated WJ-MSC secretion was quantified [24].

## Conclusions

AT-MSC, BM-MSC and WJ-MSC were successfully differentiated into adipogenic-like cells under appropriate stimulation by the culture media. Considering the behavior described *in vitro* for all three adipocyte-like cells obtained in the present work, AT- and BM-MSC would be good candidates for fat supplementation during transplantation.

## Methods

### Ethics statements

All donors signed an informed consent before tissue donation. Ethics Research Committee of the Pro-Cardiaco Hospital (Rio de Janeiro, Brazil) reviewed and approved isolation and use in research of adipose tissue-derived mesenchymal stem cells (AT-MSC; CEP: 55219/12), Wharton’s Jelly-derived mesenchymal stem cells (WJ-MSC; CEP: 336/10), and bone marrow-derived mesenchymal stem cells (BM-MSC; CEP: 473/12).

### Cell preparation and adipogenic differentiation in vitro

MSC were isolated as described previously [21]. MSC identity was confirmed by following the guidelines established by the Mesenchymal and Tissue Stem Cell Committee of the International Society for Cellular Therapy: MSC must adhere to plastic under standard conditions, express surface molecules and differentiate into osteoblasts, adipocytes and chondroblasts [22]. Cells were expanded until passage number 3, by detaching the cells using trypsin, determining the concentration by manual counting and diluting the cells to 5 × 10^5^ cells/mL; passages were performed every four days. A pool for each MSC was prepared by mixing MSC derived from four different donors so donor-dependent variability was decreased. Three replicates were performed for each assay using the same pool of cells.

Cell pools were seeded into 24-well plates (2 mL/well) at 25,000 cells/mL. Differentiation medium was replaced twice a week. It consisted of low glucose DMEM (BR30002-05, LGC, Cotia, São Paulo, Brazil) supplemented with 10% fetal bovine serum (FBS, #10-BIO-500 LGC, Cotia, São Paulo, Brazil), 1 μM dexamethasone (D4902, Sigma, St. Louis, Missouri, USA), 0.5 mM 3-isobutyl-1-methylxanthine (I7018, Sigma, St. Louis, Missouri, USA), 10 μM human insulin (Humulin-N, Eli Lilly, Indianapolis, Indiana, USA), 0.2 mM indomethacin (I7378, Sigma, St. Louis, Missouri, USA) and a penicillin/streptomycin solution (BR30110-01, LGC, Cotia, São Paulo, Brazil) at 100 U/mL and 100 μg/mL, respectively. After 3 weeks, cells were incubated with 0.5% Oil Red O solution (O0625, Sigma, St. Louis, Missouri, USA) in order to stain intracellular accumulated lipids.

### RNA extraction and qPCR

RNeasy Plus Mini kit (#74134, QIAGEN, Germantown, Maryland, USA) was used for purifying total RNA from freshly isolated cells (t = 0) or cells submitted to culture conditions that favor differentiation into adipocytes (t = 10 or 21 days). RNA concentration was determined using a Nanodrop 2000 UV–vis spectrophotometer (Thermo Scientific, Waltham, Massachusetts, USA). Purified RNA was retro-transcribed using a SuperScript VILO Mastermix (#11755250, Invitrogen, Carlsbad, California USA), according to manufacturer’s instructions. qPCR reactions were performed in an Applied Biosystems 7500 Fast Real Time PCR System using TaqMan Gene Expression Mastermix (#4369510, Applied Biosystems, Foster City, California, USA), according to manufacturer’s instructions. Pre-designed TaqMan Gene Expression assays (#4331182, Applied Biosystems, Foster City, California, USA) were used to quantify the relative expression levels of pluripotent and adipogenic genes. Assay identification numbers used in the present work are: Hs02800695_m1 for the analysis of *HPRT1* expression (reference gene), Hs03043885_g1 for the analysis of *RPL13A* expression, Hs01053049_s1 for the analysis of *SOX2* expression, Hs00999634_gH for the analysis of *POU5F1* expression, Hs00972656_m1 for the analysis of *TERT* expression, Hs01115513_m1 for the analysis of *PPARG* expression, Hs00605917_m1 for the analysis of *ADIPOQ* expression and Hs00269972_s1 for the analysis of *CEPBA* expression. Data was analyzed using the 2^(−delta delta Ct) method as previously described by Livak and colleagues [23]; relative gene expression from differentiated cells is calculated relative to the expression of the same gene in non-differentiated cells (growth in parallel during 21 days under control media).

### Cytokine, growth factor and extracellular matrix quantification

We used commercial Luminex and ELISA kits for quantifying cell supernatant concentration of 49 different cytokines, growth factors and extracellular matrix-related proteins. Human Cytokine 30-plex Assay (Invitrogen, Carlsbad, California USA) was used for the quantification of chemokines, pro-and anti-inflammatory cytokines and growth factors (GM-CSF, G-CSF, IL-1β, IL-1RA, IL-2, IL-2R, IL-4, IL-5, IL-6, IL-7, IL-8, IL-10, IL-12p40/p70, IL-13, IL-15, IL-17, TNF-α, IFN-α, IFN-γ, eotaxin, IP-10, MCP-1, MIG, MIP-1α, MIP-1β, RANTES, EGF, bFGF, HGF, VEGF). TGF-β1, TGF-β2 and TGF-β3 were quantified using the Fluorokine MAP TGF-β Multiplex Kit (R&D, Minneapolis, Minnesota, USA). Human Angiogenesis Fluorokine Multi Analyte Profiling Kit (R&D, Minneapolis, Minnesota, USA) was customized for the quantification of angiogenic growth factors (VEGF-D, endostatin, aFGF, thrombospondin-2, angiopoietin-1, angiogenin, PDGF-AA, PDGF-BB and PlGF). Matrix metalloproteinases (MMP-1, −3, −7, −8 and −13) were quantified using the Fluorokine MAP Human MMP kit (R&D, Minneapolis, Minnesota, USA) and Milliplex MAP Human IGF-1 Single Plex Kit (Millipore, Billerica, Massachusetts, USA) was used for IGF-1 quantification. PDGF-AB was quantified using an ELISA kit Quantikine hPDGF-AB ELISA (R&D, Minneapolis, Minnesota, USA). Extracellular matrix components heparan sulphate, aggrecan, decorin, elastin, laminin, perlecan, fibronectin and collagens type I, II, III and IV were quantified in cell culture supernatants using commercial ELISA kits (E0623h, E91908Hu, E92127Hu, E91337Hu, E90082Hu, E82748Hu, E90037Hu, E90571Hu, E90572Hu, E90176Hu and E90180Hu, respectively; USCN, Wuhan, Hubei, China). Procedures were performed according to manufacturer’s instructions in all cases.

We analyzed cell supernatants obtained from the last differentiation culture media replacement, just before staining for lipid accumulation. Since cell concentration was different for the three cell types at the end of the adipogenic differentiation, protein concentrations were normalized by final cell concentration (determined by manual counting using Trypan Blue after detaching the cells using trypsin) and incubation time since last culture media replacement, expressing the results in pg/10^6^ cells/day (considering final cell concentration, since protein content refers to the last 3 days of the culture). Results were expressed as mean ± standard deviation for 3 replicates.

### Statistical analysis

Statistical significance was assessed by one-way non-parametric ANOVA followed by Bonferroni post-hoc test to compare all pairs of data. P-values < 0.05 were considered statistically significant. Statistical analysis was performed using the Prism 5.00 Software (GraphPad Software Inc., San Diego, California, USA).

## Abbreviations

*ADIPOQ*: Adiponectin
AT-MSC: Adipose tissue-derived MSC
BM-MSC: Bone marrow-derived MSC
*sCEPBA*: CCAAT/enhancer-binding protein alpha
DMEM: Dulbecco’s Modified Eagle’s Medium
FBS: Fetal bovine serum
*HPRT1*: Hypoxanthine phosphoribosyltransferase 1
MSC: Mesenchymal stromal cells
nd: Not determined
*Oct4*: Octamer-binding transcription factor 4
*POU5F1*: POU domain, class 5, transcription factor 1
*PPARG*: Peroxisome proliferator-activated receptor gamma
qPCR: Quantitative real time polymerase chain reaction
*RPL13A*: 60S ribosomal protein L13A
*SOX2*: SRY (Sex Determining Region Y)-Box 2
*TERT*: Telomerase reverse transcriptase
WJ-MSC: Wharton’s Jelly-derived MSC
